# Exploration of an alternative reconstructed individual patient data-based approach for budget impact analysis of anticancer drugs

**DOI:** 10.1186/s12962-023-00447-7

**Published:** 2023-06-11

**Authors:** Yue Ma, Jiting Zhou, Yuxin Ye, Aixia Ma, Hongchao Li

**Affiliations:** 1 School of International Pharmaceutical Business, Jiangning District, China Pharmaceutical University, 639 Longmian Avenue, Nanjing, 211198 Jiangsu China; 2 Center for Pharmacoeconomics and Outcomes Research of China Pharmaceutical University, Nanjing, 211198 Jiangsu China

**Keywords:** Budget impact analysis, Anticancer drugs, Duration of treatment, Reconstructed individual patient data, Economic evaluation, Reimbursement

## Abstract

**Background:**

The duration of treatment (DOT) of the initial intervention and subsequent treatment is the key to determining the accuracy of anticancer-drug budget impact analysis (BIA) calculations. However, existing studies only use simple assumptions as a proxy for DOT, resulting in a high degree of bias.

**Objectives:**

To enhance the accuracy and reliability of anticancer-drug BIA and solve the problem regarding DOT, we propose an alternative individual patient data (IPD)-based approach that reconstructs IPD from the published Kaplan Meier survival curves to estimate DOT.

**Methods:**

We developed a four-step methodological framework for this new approach, taking the use of pembrolizumab in treating microsatellite-instability–high (MSI-H) advanced colorectal cancer as an example: (1) reconstructing the IPD; (2) calculating the total DOT of the initial intervention and subsequent treatment for each patient; (3) assigning a randomized time and DOT; and (4) multiple replacement sampling and calculation of the mean value.

**Results:**

Using this approach, the average DOT for the initial intervention and subsequent treatment in each year of the BIA time horizon can be calculated and used to calculate the resources consumed and costs in each year. In our example, the average DOT for the initial intervention with pembrolizumab from the first to the fourth year was 4.90, 6.60, 5.24, and 5.06 months, respectively, while the average DOT for subsequent treatment was 0.75, 2.84, 2.99, and 2.50 months, respectively.

**Conclusions:**

The reconstructed IPD-based approach can improve the accuracy and reliability of anticancer-drug BIA compared with conventional methods, and can be widely used, especially for anticancer drugs with excellent efficacy.

**Supplementary Information:**

The online version contains supplementary material available at 10.1186/s12962-023-00447-7.

## Introduction

Budget impact analysis (BIA) is an economic assessment tool that is used to evaluate the financial affordability of adopting a new health-care intervention or technology in a specific health-care setting or system given inevitable resource constraints [1, 2]. In recent years, BIA has gained popularity in numerous jurisdictions, including low- to upper-middle income countries with constrained budgets such as China and India, as well as high-income countries like the United States, England, and Australia, it is widely recognized as a valuable tool that can be used to support budget holders in decision-making, providing an essential complement to cost-effectiveness analysis (CEA) in efforts to optimize medical resource allocation [3–6]. It follows that the accuracy and robustness of BIA results are highly influential for budget holders and their decision-making.

Cancer is a group of diseases involving abnormal cell growth with the potential to invade or spread to other parts of the body [7] that has become one of leading causes of death in many countries. The global burden of disease database has recorded a continuous increase in the global cancer incidence and mortality rates over recent years. For example, the numbers of deaths from colon and rectal cancer, cervical cancer, breast cancer, and tracheal, bronchial, and lung cancer increased from 837,376, 233,890, 550,621, and 1,631,778, respectively, in 2009 to 1,085,797, 280,479, 700,660, and 2,042,640, respectively, in 2019 [8]. To improve the longevity and quality of life of cancer patients, an increasing number of innovative anticancer drugs have been approved globally in recent years [9–12]. BIA results provide important evidence for the marketing and pricing of these drugs, mainly by assisting payers and decision-makers to determine whether to list them for national or commercial reimbursement [13].

Numerous elements need to be considered in BIA, including the study perspective, target population, market scenarios, time horizon, market share, costs, computing framework, uncertainty and scenario analyses, validation and data sources, and hierarchy [1, 2, 6]. Of these elements, the most important are costs, which have a significant direct influence on BIA results. The costs of the initial intervention and subsequent treatment can be calculated based on their unit price and the amount used in the target population, with the latter being determined by the duration of treatment (DOT). Although the DOT is crucial regarding BIA results, it has usually simply been assumed as an overall parameter in most existing BIAs for anticancer drugs. The DOT of the initial intervention was simply assumed to be the median progression free survival (PFS) and the DOT of subsequent treatment was simply assumed to be the difference between the median overall survival (OS) and the median PFS [14]. However, these simple assumptions so not solve several problems related to BIA for anticancer drugs with excellent efficacy, for example, (1) a single DOT parameter cannot reflect the randomness of the treatment time points of individual patients (although not all patients receive their initial intervention on 1 January, 12 months is usually assumed to be the upper limit of the DOT for each year, which might overestimate the DOT in a given year), (2) when the median PFS or median OS reported in the literature is more than 12 months, we cannot calculate the costs of both the initial intervention and subsequent treatment in the same year, and (3) we cannot estimate the costs of patients remaining from the previous year in a given year. These problems introduce bias to the BIA, and thus uncertainty to related medical decision-making. However, there have been no studies exploring these issues in depth and proposing appropriate solutions.

Thus, in this study, we develop an alternative reconstructed individual patient data (IPD)-based approach for BIA for anticancer drugs and calculate the DOT of the initial intervention and subsequent treatment using information on individual patients instead of simple assumptions. This innovative approach solves the abovementioned BIA problems to some extent, which is necessary to enhance the accuracy and reliability of BIA results and subsequent medical decision-making.

## Methods

### Overview of the reconstructed IPD-based approach

In an ideal situation, we can calculate the average DOT of the initial intervention and subsequent treatment using IPD for the entire target population considered in the BIA. Using IPD, we know when a specific patient receives the initial intervention, when subsequent treatment changes because of disease progression, and when treatment ends because of death in each year. Thus, we attempted to acquire IPD for all interventions to be evaluated.

**Figure1:**
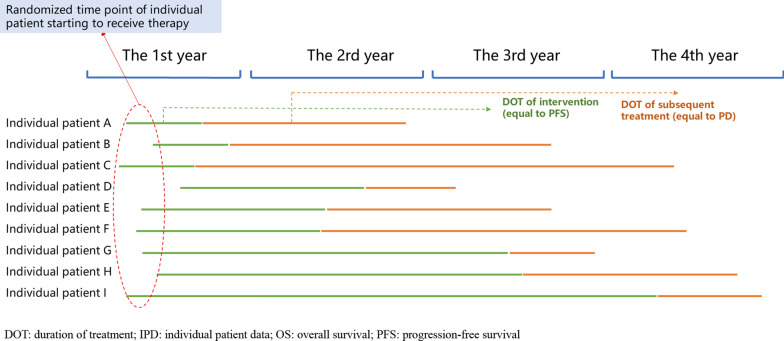
The DOT of individual patients in each year determined by IPD

Two categories of intervention need to be considered in BIA: new interventions and current interventions. Regarding BIA for anticancer drugs, in most cases we were only able to obtain the Kaplan–Meier (KM) curves for all interventions from published articles, which used aggregated data instead of IPD. Thus, to obtain IPD, we used the algorithm developed by Guyot et al. to reconstruct the KM curve data based on the PFS and OS curves [15]. With reconstructed IPD including the duration of PFS and duration of progression of disease (PD, calculated as OS minus PFS), we assigned a randomized start time of treatment to each individual patient, and then assigned a DOT of the initial intervention (equal to PFS) and a DOT of subsequent treatment (equal to PD) to each year. The DOT for individual patients in each year as determined by IPD is shown in Fig. 1. At this point, the IPD sample size was equal to the clinical trial sample size. Next, we performed multiple replacement sampling (e.g., 1000 or consistent with the number of new cases in each year of the target population) to simulate the DOT of the initial intervention and subsequent treatment for each individual patient in each year, or to calculate the average DOT of the initial intervention and subsequent treatment in each year, which can be used to calculate the initial intervention and subsequent treatment costs based on unit price and dosage.

**Figure2:**
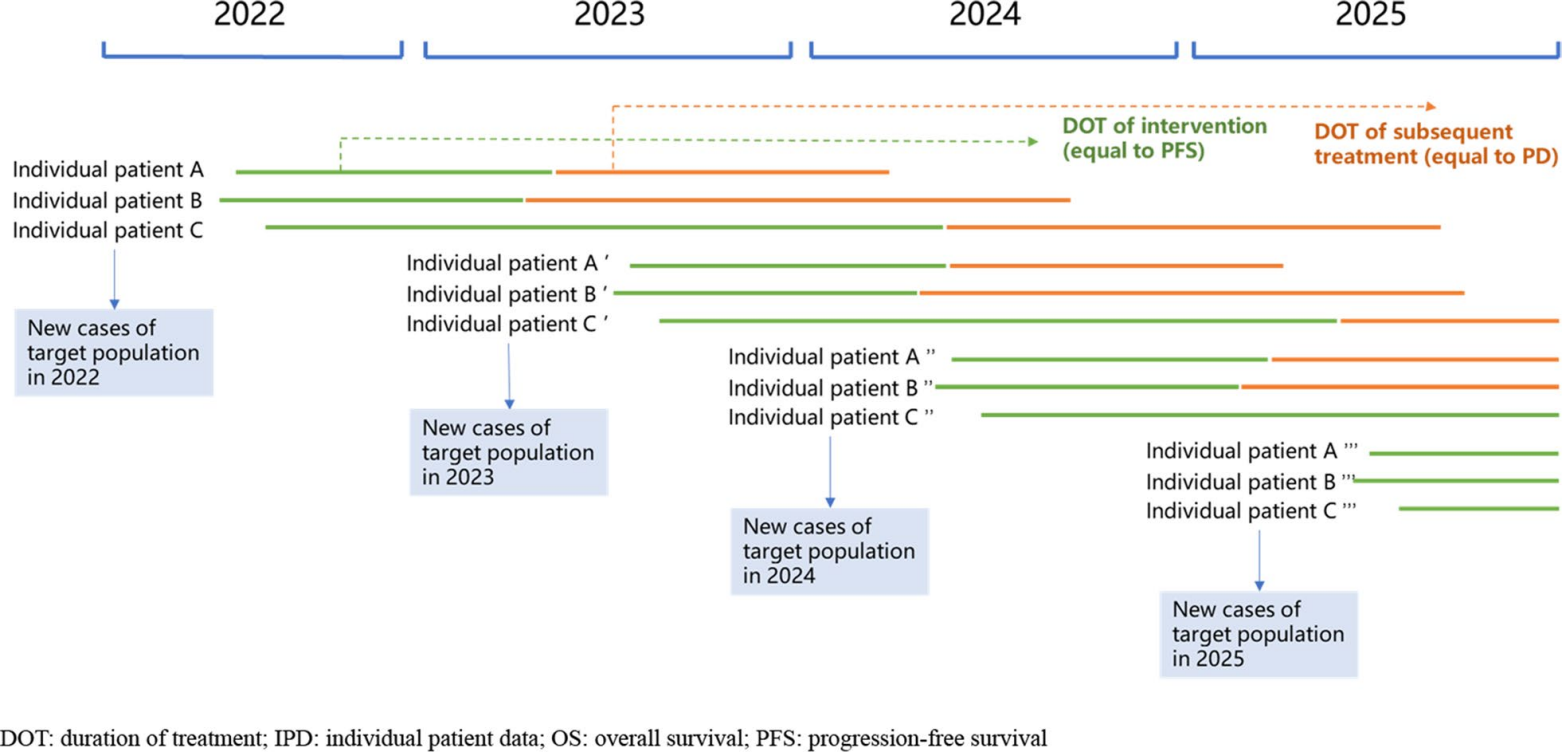
The DOT of new cases of target population in each year determined by IPD

**Fig. 3:**
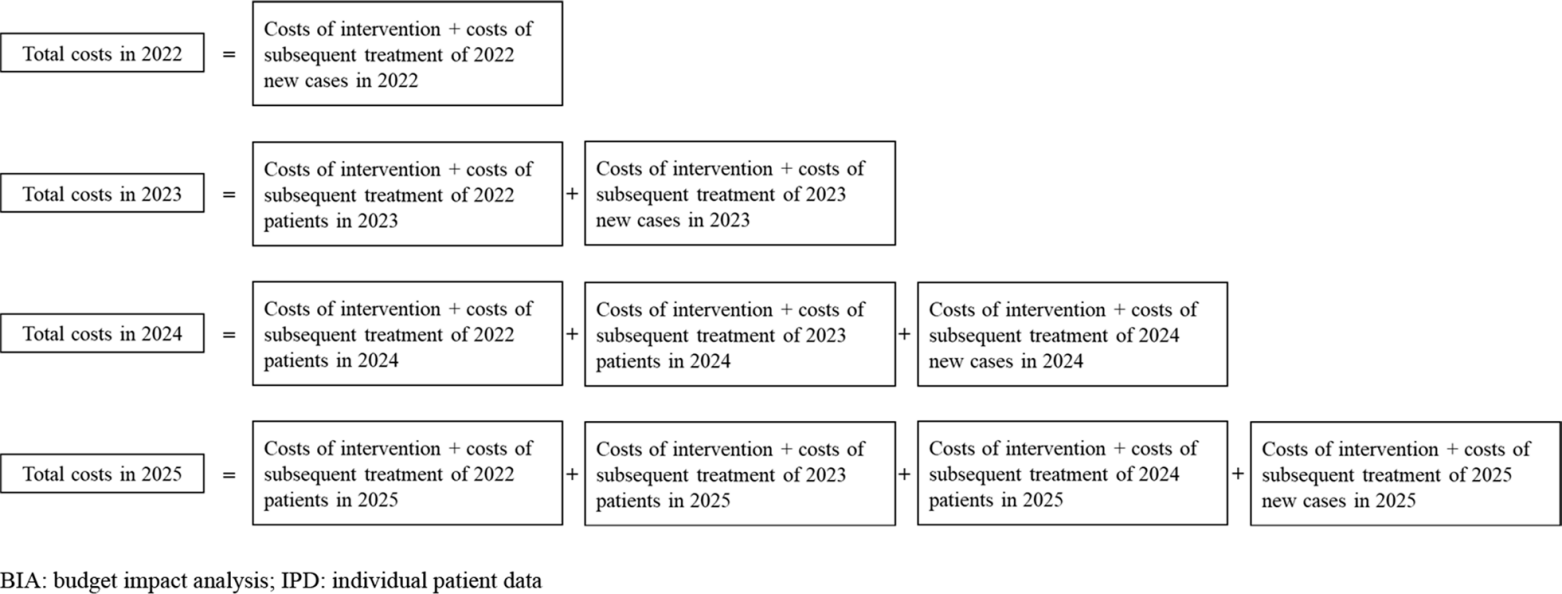
The cost calculating framework by reconstructed IPD-based approach for anticancer-drug BIA

In accordance with the budgeting process and periods of the budget holder (e.g., monthly, quarterly, or annually), BIAs are commonly presented for time horizons of one to five years, with the results presented for each budget period after the new intervention is covered [2]. Therefore, taking a four-year time horizon (2022–2025) as an example, the treatment costs of patients who are new cases in 2022 and still alive in 2023, 2024 and 2025 need to be accounted for in all four years, and the treatment costs of patients who are new cases in 2023 and still alive in 2024 and 2025 need to be accounted for in all three years, and so on. In this example, the DOT of new cases in the target population in each year as determined by IPD is shown in Fig. 2, while the cost calculation framework is shown in Fig. 3. Using this approach, we can calculate the medical resources consumed and the related costs of the target population not only in the initial year for new cases but also in subsequent years while the patients remain alive.

### Methodological framework

To present the process in sufficient detail to enable the study to be replicated by others, we used pembrolizumab in the treatment of microsatellite-instability–high (MSI-H) advanced colorectal cancer (CRC) as an example to describe the steps in our reconstructed IPD-based approach [16, 17].

### Step 1 reconstructing IPD

Pembrolizumab is a type of PD-1 inhibitor, and KM curves of the PFS and OS when it is used to treat patients with MSI-H advanced CRC have been reported in previous studies [16]. A total of 153 patients were enrolled in a clinical trial of pembrolizumab [16]. Because programmed death 1 (PD-1) blockade is a highly effective form of therapy for patients with MSI-H metastatic CRC, the PFS and OS were not mature during the follow-up period using pembrolizumab (the median PFS was 16.5 months and more than 40% of patients were in PFS at the end of follow-up, while the median OS was not reached). Reconstructing IPD from immature KM curves will lead to many censoring patients, thereby underestimating the DOT. Hence, we reconstructed IPD using two sub-steps.

Firstly, we used DigitizeIt software (http://www.digitizeit.de/) to extract x-axis coordinates and y-axis coordinates based on the PFS and OS reported in the published studies, and then reconstructed IPD based on the extracted data for the two curves [15]. We called that data the first-reconstructed IPD. Using the first-reconstructed IPD, we fitted standard parametric survival models for PFS and a mixture cure model for OS with different parametric distributions including exponential, gamma, Gompertz, Weibull, log-logistic and log-normal distribution, and then determined the suitability of fitted models by visual inspection and Akaike’s Information Criterion (AIC)/Bayesian Information Criterion (BIC) tests to choose the most suitable model [18]. Because of the immaturity of the original KM curves, the fitted models based on the first-reconstructed IPD were also immature. Thus, we referred to the experience of NICE guidance, introducing and applying a twofold increase in the mortality rate to the fitted model to extrapolate mature survival curves (more than 99% patients either progressed or died) [19]. The all-cause mortality rate by age was obtained from the World Health Organization mortality database [20].

Secondly, using mature curves for PFS and OS, we reconstructed the IPD again following the same method. We called that the second-reconstructed IPD, which was free of censoring problems, and thus able to be used to calculate the DOT of pembrolizumab and subsequent treatment in each year. All of the abovementioned statistical analyses were performed using the R 4.1.2 software package and the reconstructed IPD were recorded in Microsoft Excel 2019.

### Step 2 calculating the total DOT of the initial intervention and subsequent treatment for each patient

After reconstructing the IPD, we obtained the PFS and OS of all patients and ranked them in ascending order respectively to obtain PFS and OS of each patient. It must be emphasized that there is an assumption that patients who progressed early died early. Then, we calculated the difference between the OS and PFS for each patient to obtain the PD for each patient. The PFS was used as the total DOT of the initial intervention with pembrolizumab and the PD was used as the total DOT for subsequent treatment of each patient.

### Step 3 assigning a randomized time and DOT

Based on the randomness of the time of receiving treatment for each patient, we randomly generated a treatment start time in the first year for each patient using Microsoft Excel 2019. Using the randomized start time and the final day of the year (31 December), we easily calculated the residual months in the first year for each patient. Then, based on the randomized start time in the first year, the residual months in the first year, the numbers of months in one year (12), the total DOT of the initial intervention with pembrolizumab and the total DOT for subsequent treatment, we assigned a DOT to each year for each patient. The time horizon for our example was four years, therefore the assigned DOT included the DOT of the initial intervention with pembrolizumab and the DOT for subsequent treatment in each of the first, second, third, and fourth years. The schematic diagram of the DOT assignment process is shown in Fig. 1.

### Step 4 multiple replacement sampling and calculation of the mean value

In an effort to improve the robustness of the calculated DOT, we performed 1000 times randomized replacement sampling using IPD for all 153 subjects. After sampling, we obtained 1000 sets of IPD and the corresponding DOT of the initial intervention with pembrolizumab and subsequent treatment in each year. Then, we calculated the average DOT of the initial intervention with pembrolizumab and subsequent treatment in the first, second, third, and fourth years, respectively.

The unit for DOT was one month, and using information on the unit price and dosage of pembrolizumab and subsequent treatments, we were able to calculate the quantity of pembrolizumab, subsequent treatments, and other medical resources used, and their costs in each year. Using the calculation framework shown in Figs. 2 and 3 and the costs and numbers of new cases in the target population in each year, all costs were able to be calculated across the BIA time horizon.

## Results

### Reconstructed IPD and total DOT

**Fig. 4:**
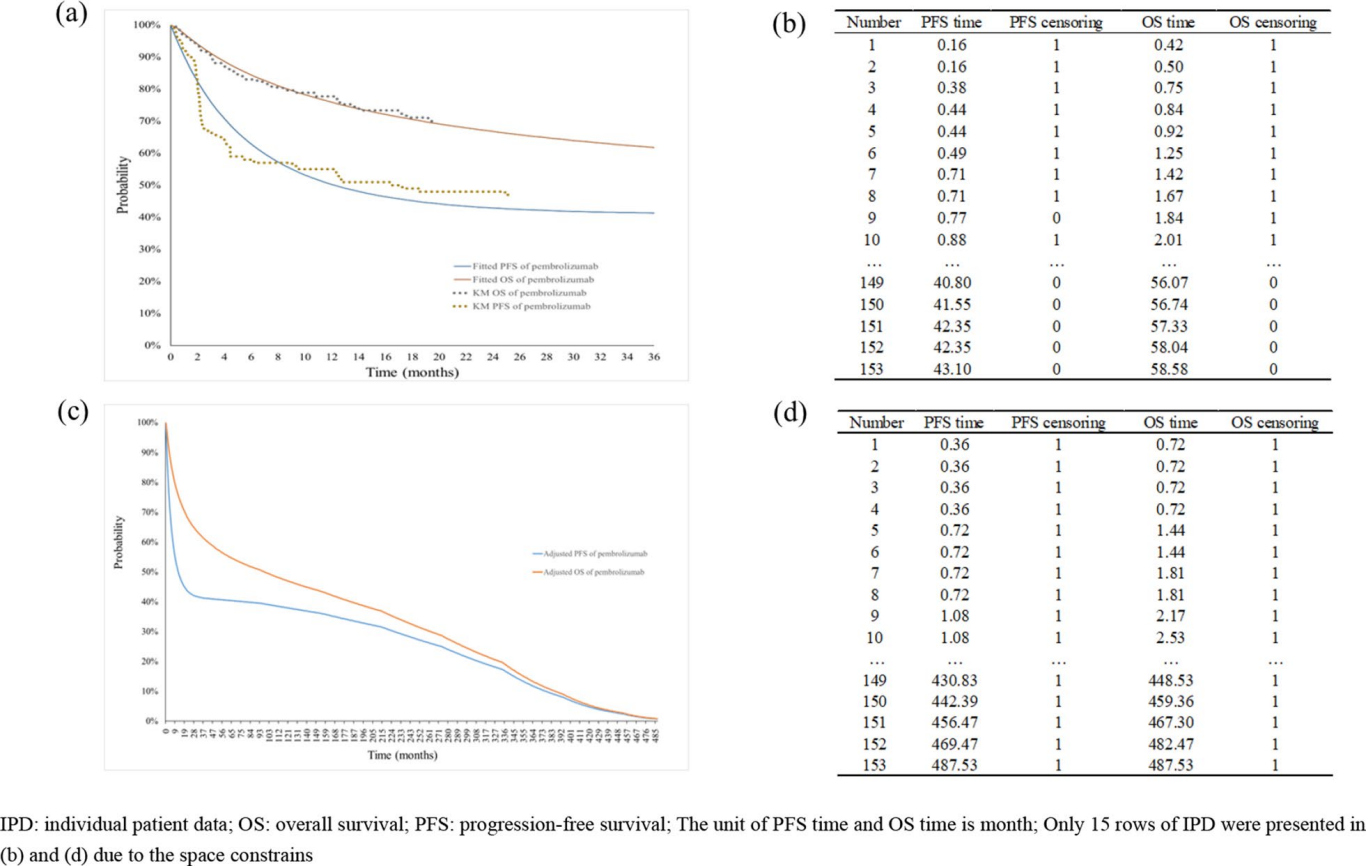
Survival curves and reconstructed IPD of pembrolizumab, **a** is reproduced Kaplan–Meier curves and fitted survival curves, **b** is first-reconstructed IPD, **c** is adjusted mature survival curves and **d** is second-reconstructed IPD

The KM curves for pembrolizumab used in treating MSI-H advanced CRC in our illustrative example are shown in Fig. 4a, and the first-reconstructed IPD that were reconstructed from the original KM curves are shown in Fig. 4b. Because of the immaturity of the original KM curves, there were more than half censoring patients (i.e., allocated a value of 0) in the first-reconstructed IPD.

Based on the first-reconstructed IPD, six standard parametric models for PFS and six mixture cure models for OS were fitted, and the Gompertz distribution for PFS and the log-normal distribution for OS were assessed to be the most suitable models based on visual inspection and AIC/BIC tests. Given the immaturity of the original curves, these fitted curves were also immature. After introducing a twofold increase in the mortality rate to the fitted model, we obtained the adjusted mature survival curves, which are shown in Fig. 4c. The median PFS and median OS of the adjusted survival curves were 12.1 months and 95.2 months, respectively.

Based on the adjusted survival curves, the second reconstruction was performed. The second-reconstructed IPD are shown in ascending order in Fig. 4d. There were no censoring patients in the second-reconstructed IPD, which were used to represent the actual PFS and OS of the patients. The PFS was used as the DOT of the initial intervention with pembrolizumab and the PD (calculated as OS minus PFS) was used as the DOT for subsequent treatments considered in the BIA for each patient.

### DOT assignment and multiple replacement sampling

The results after assigning a randomized start time to each patient, assigning the DOT of the initial intervention with pembrolizumab and subsequent treatment to each year, and multiple replacement sampling are presented in Table 1. Because of space limitations, only 20 rows of data are shown.

**Table 1:** The DOT assignment and multiple replacement sampling results

Sampling number	Patient number from reconstructed IPD	PFS time	PFS censoring	OS time	OS censoring	PD time	Randomized start time of receiving treatment	Date of the end of year	Residual months in the first year	DOT of intervention in the 1st year	DOT of subsequent treatment in the 1st year	DOT of intervention in the 2nd year	DOT of subsequent treatment in the 2nd year	DOT of intervention in the 3rd year	DOT of subsequent treatment in the 3rd year	DOT of intervention in the 4th year	DOT of subsequent treatment in the 4th year
1	114	260.74	1	287.82	1	27.08	01/06	12/31	11.97	11.97	0.00	12.00	0.00	12.00	0.00	12.00	0.00
2	12	1.08	1	2.89	1	1.81	08/26	12/31	4.23	1.08	1.81	0.00	0.00	0.00	0.00	0.00	0.00
3	142	378.10	1	402.66	1	24.56	05/17	12/31	7.60	7.60	0.00	12.00	0.00	12.00	0.00	12.00	0.00
4	117	278.79	1	298.66	1	19.86	02/01	12/31	11.10	11.10	0.00	12.00	0.00	12.00	0.00	12.00	0.00
5	11	1.08	1	2.53	1	1.44	07/24	12/31	5.33	1.08	1.44	0.00	0.00	0.00	0.00	0.00	0.00
6	150	442.39	1	459.36	1	16.97	02/13	12/31	10.70	10.70	0.00	12.00	0.00	12.00	0.00	12.00	0.00
7	109	230.04	1	270.13	1	40.09	01/20	12/31	11.50	11.50	0.00	12.00	0.00	12.00	0.00	12.00	0.00
8	133	353.19	1	362.22	1	9.03	12/22	12/31	0.30	0.30	0.00	12.00	0.00	12.00	0.00	12.00	0.00
9	145	399.41	1	417.11	1	17.70	03/22	12/31	9.47	9.47	0.00	12.00	0.00	12.00	0.00	12.00	0.00
10	144	392.91	1	412.05	1	19.14	05/12	12/31	7.77	7.77	0.00	12.00	0.00	12.00	0.00	12.00	0.00
11	150	442.39	1	459.36	1	16.97	02/13	12/31	10.70	10.70	0.00	12.00	0.00	12.00	0.00	12.00	0.00
12	19	1.81	1	4.69	1	2.89	01/28	12/31	11.23	1.81	2.89	0.00	0.00	0.00	0.00	0.00	0.00
13	82	15.89	1	123.51	1	107.62	03/17	12/31	9.63	9.63	0.00	6.26	5.74	0.00	12.00	0.00	12.00
14	112	248.82	1	282.77	1	33.95	06/27	12/31	6.23	6.23	0.00	12.00	0.00	12.00	0.00	12.00	0.00
15	11	1.08	1	2.53	1	1.44	07/24	12/31	5.33	1.08	1.44	0.00	0.00	0.00	0.00	0.00	0.00
…	…	…	…	…	…	…	…	…	…	…	…	…	…	…	…	…	…
996	19	1.81	1	4.69	1	2.89	01/28	12/31	11.23	1.81	2.89	0.00	0.00	0.00	0.00	0.00	0.00
997	80	14.45	1	112.67	1	98.23	11/29	12/31	1.07	1.07	0.00	12.00	0.00	1.38	10.62	0.00	12.00
998	88	24.92	1	159.62	1	134.70	02/03	12/31	11.03	11.03	0.00	12.00	0.00	1.88	10.12	0.00	12.00
999	41	3.97	1	15.17	1	11.20	12/29	12/31	0.07	0.07	0.00	3.91	8.09	0.00	3.10	0.00	0.00
1000	13	1.44	1	3.25	1	1.81	12/06	12/31	0.83	0.83	0.00	0.61	1.81	0.00	0.00	0.00	0.00
									Average	4.90	0.75	6.60	2.84	5.24	2.99	5.06	2.5

*DOT* duration of treatment; *IPD* individual patient data; *OS* overall survival; *PFS* progression-free survival

The first and second columns show the sample numbers from 1 to 1000 and the patient numbers from 1 to 153 for the reconstructed IPD, respectively. Based on the randomized time of receiving treatment and the residual months in the first year for each patient, shown in the fifth and seventh columns, respectively, the DOT of the initial intervention with pembrolizumab (equal to the PFS shown in the third column) and the DOT for subsequent treatment (equal to the PD shown in the fourth column) are shown in columns eight to 15, including the DOT of the initial intervention with pembrolizumab and the DOT for subsequent treatment in each of the first four years.

Taking patient number 27 as an example, the PFS and PD were 2.53 and 4.69 months, respectively. With a randomized start time of 9 February, the patient had 10.83 months left in the first year. Because 7.22 months (PFS of 2.53 months plus PD of 4.69 months) is less than 10.83 months, the DOT of the initial intervention with pembrolizumab was 2.53 months in the first year and the DOT for subsequent treatment was 4.69 months in the first year, while the DOT of the initial intervention with pembrolizumab and the DOT for subsequent treatment in the subsequent three years were all 0 months.

The average DOT for the 1000 times sampling is shown in the final row in Table1. The average DOT of the initial intervention with pembrolizumab in the first to the fourth year was 4.90, 6.60, 5.24, and 5.06 months, respectively, while the average DOT for subsequent treatment in the first to the fourth year was 0.75, 2.84, 2.99, and 2.50 months, respectively.

## Discussion

With the continuing progress of precision medicine and pharmacy in recent years, a growing number of innovative anticancer drugs such as immune checkpoint inhibitors, antibody–drug conjugates, and gene therapies have been approved globally [21–23]. BIA provides valuable evidence supporting medical decision-making regarding pricing and reimbursement, and thus is widely used by decision-makers and payers to estimate the affordability of these drugs [24, 25]. In essence, BIA is a type of cost calculator, and thus the quantity of anticancer drugs used in the initial intervention and subsequent treatment as part of a therapeutic regimen is essential. Ideally, to calculate the quantity of drugs used for the entire target population in BIA, we need the DOT of interventions with the anticancer drug (including new interventions and current interventions) and subsequent treatment for each individual patient. However, simple assumptions such as using the median PFS and the median OS minus the median PFS as proxies for the DOT of the intervention and subsequent treatment, respectively, have been applied in most existing anticancer-drug BIAs [14]. For example, Westerink et al. used the median PFS as a proxy for the DOT of afatinib for first-line EGFR-mutant non-small-cell lung cancer [26], while Mennini et al. used the same assumption in relation to the use of cetuximab for recurrent and/or metastatic head and neck squamous cell cancer [27]. These assumptions reduce the quality of anticancer-drug BIA results and related decision-making. In addition, in accordance with the budgeting process and periods used by most budget holders, a budget period of a single year is commonly used [2]. But for the conventional method, it is difficult to calculate the DOT of subsequent treatment in a specific year when median PFS is over than 12 months. In response to these problems in relation to existing BIAs for anticancer drugs, we propose an alternative in the form of a reconstructed IPD-based approach. Furthermore, the objective of this study aligns with some previous researches’ efforts, namely using K-M curves to enhance the accuracy of predicted economic results for innovative drugs, for better value-based evaluation and related decision-makings [28, 29].

Although IPD related to the use of anticancer drugs are helpful for calculating the DOT, it is not easy to obtain primary data related to all of the drugs to which BIA is applied. Usually, researchers can only obtain aggregated data from KM curves in published articles. Thus, to obtain IPD, we used the reconstructed IPD method proposed by Guyot [15]. This method has been the most widely accepted and used reconstruction method in previous economic evaluation studies (e.g., CEA and cost-utility analysis) since it was proposed. In our study, it was applied to BIA in the first step and used to estimate the total DOT of the initial intervention and subsequent treatment for each patient.

To take the randomness of the treatment start time into account, we assigned a randomized start time to the IPD for each patient. This is the first time that a randomized start time has been considered in relation to BIA, but was considered necessary to ensure the accuracy of the results. If the randomized start time had not been considered, the conventional method would have assumed that all new cases were treated at the beginning of each year, leading to overestimation of the DOT and related costs in a given year. In addition, it would have resulted in underestimation of the DOT in a given year of patients who were new cases in previous years. In addition, the scope of the randomized start time can be adjusted based on the actual scenario. For example, it can be set from August to December in the first year if the initial intervention is approved in August.

Using the total DOT from IPD and randomized start times, we assigned a DOT for the initial intervention and subsequent treatment to each year in the time horizon. Using this method, no matter how long the PFS is, we can calculate the average DOT for both the initial intervention and subsequent treatment in the same year. Subsequent treatment costs have a significant influence on anticancer-drug BIA results, and thus budget holders, because most cancers are chronic diseases and cancer patients will change treatment regimens as the disease progresses [14]. The conventional method cannot consider subsequent treatment in a given year once the median PFS exceeds 12 months. Therefore, our reconstructed IPD-based approach can improve the accuracy and reliability of BIA results, especially for anticancer drugs with excellent efficacy.

To ensure the robustness of our results, we performed 1000 times multiple replacement sampling and calculated the mean DOT values for each year, which were then used as parameters in the BIA model to calculate costs. The sampling frequency can be set to reflect the number of new cases in the target population each year, and thus the costs for each patient in each year can be calculated. The sum of the costs of all patients can then be calculated, which is the total cost of the target population considered in the BIA. Researchers can also consider using the second calculation method when the target population is small. It should be noted that while the amount of anticancer drugs used is primarily determined by the DOT, other factors, including patient adherence, physician compliance with guidelines, and patient perception of risk, may also impact their amount in treatments of different cancers. Therefore, when utilizing this innovative approach to generate BIA evidence for decision-making purposes, it is also necessary to consider other important factors that affect drug amount in addition to DOT.

There are some limitations to the proposed approach. The main source of bias in this approach is the use of the reconstructed IPD method. Firstly, the original KM curves represent pooled data from different covariates that might affect survival, and the reconstructed IPD based on those KM curves are unable to consider these covariates. Furthermore, the original KM curves are mainly from clinical trials with low external validity, which is not the same as having real-world IPD. These issues might lead to bias in the DOT estimation for the target population. Secondly, the accuracy of the DOT estimation depends on the maturity of the KM curves. If the sample size on which the published KM curves are based is small or the duration of follow-up is short, there is already considerable uncertainty regarding the original curves, which will tend to bias the reconstructed IPD, and thus the DOT estimation. In addition, for anticancer drugs with excellent efficacy, the published KM curves usually do not reach the median PFS and median OS, and thus we need to extrapolate mature curves by using the parametric survival model and applying other assumptions (e.g., increasing the risk of death), and even reconstructing the IPD using two steps. Although these methods and assumptions address the problems caused by immature KM curves and censoring of reconstructed IPD, they also increase the uncertainty of the DOT estimation. We hope that researchers will validate this proposed approach in future studies based on real-world data, thereby confirming its practicability. Thirdly, the new approach is more time-consuming than the conventional method, and researchers using the proposed approach might also need to be trained in extracting data and reconstructing IPD. In our experience, we recommend that at least two researchers should collaborate in implementing this approach when numerous interventions need to be considered in BIA. At the same time, in certain exceptional circumstances such as conducting BIA for anticancer drugs on patients with very short survival times (e.g., advanced pancreatic cancer patients), researchers can weigh the trade-off between BIA accuracy and technical support when deciding whether to adopt the new approach or the conventional methods. Given that these patients' PFS and PD times are so short, the time-consuming new approach offers limited advantages in reducing uncertainty.

Finally, it is necessary to emphasize that because BIA is a type of predictive tool, it is inherently highly uncertain, and its results cannot be guaranteed to be completely accurate [30]. Our proposed approach does not suggest that all BIAs need to be highly elaborate, but aims to solve the problems related to DOT, which obviously affect anticancer-drug BIA results, in an effort to improve the reliability of the results and the related medical decision-making.

## Conclusion

For anticancer-drug BIA, the DOT of both the initial intervention and subsequent treatment are highly influential on cost calculations. However, previous studies have made simple assumptions as a proxy for the DOT, resulting in a high degree of bias. Thus, we propose an alternative reconstructed IPD-based approach in an effort to solve this problem. The proposed approach is based on KM curves reported in published articles, and we use an example to illustrate the four detailed implementation steps. The complete reconstructed IPD and calculation process is presented in an Excel template in the Additional file 1. The proposed approach will improve the accuracy and reliability of anticancer-drug BIA and related medical decision-making.

## Supplementary Information

**Additional file 1: Appendix 1.** First-reconstructed IPD based on pembrolizumab surival curves in MSI-H advanced colorectal cancer.

## Data Availability

All data generated or analysed during this study are included in this published article [and its Additional files].
